# Retraction: Lemierre’s Syndrome: A Case of Life-Threatening Infection From Family Medicine Practice

**DOI:** 10.7759/cureus.r118

**Published:** 2024-01-25

**Authors:** Sultan O Gohal, Ishtiyaq M Alsubhi, Essa A Alharbi, Hashim E Alkhalaf, Bandar H Alnefaie, Raghad A Althomali, Bayan A Hasan, Mada A Alsadi, Assaf H Alamri, Abdulrahman A Alharbi, Meshal Y Almanea, Ftoon S Almarshood, Saleh A Almoallem, Afaf F Albogami, Faisal Al-Hawaj

**Affiliations:** 1 College of Medicine, Batterjee Medical College, Jeddah, SAU; 2 Department of General Practice, King Salman Specialist Hospital, Hail, SAU; 3 College of Medicine, Northern Border University, Arar, SAU; 4 College of Medicine, King Faisal University, Hofuf, SAU; 5 College of Medicine, King Saud bin Abdulaziz University for Health Sciences, Riyadh, SAU; 6 College of Medicine, Arabian Gulf University, Manama, BHR; 7 College of Medicine, Jordan University of Science and Technology, Irbid, JOR; 8 Department of Otolaryngology, King Abdulaziz Hospital, Makkah, SAU; 9 College of Dentistry, Riyadh Elm University, Riyadh, SAU; 10 College of Medicine, Alfaisal University, Riyadh, SAU; 11 College of Medicine, Jouf University, Al-Jawf, SAU; 12 College of Medicine, Taif University, Taif, SAU; 13 College of Medicine, Imam Abdulrahman Bin Faisal University, Dammam, SAU

The Editors-in-Chief have retracted this article. Concerns were raised regarding the identity of the authors on this article. Specifically, Faisal Alhaway and Malak Shammari have stated that they were added to this article without their knowledge or approval. The identity of the other authors could also not be verified. As the appropriate authorship of this work cannot be established, the Editors-in-Chief no longer have confidence in the results and conclusions of this article.

